# Relationship between Er,Cr:YSGG laser power and surface roughness of lased radicular dentin

**DOI:** 10.15171/joddd.2018.013

**Published:** 2018-06-20

**Authors:** Zohre Sadat Hosseinipour, Maryam Pirmoradian-najafabadi, Sima Shahabi

**Affiliations:** ^1^Department of Pediatric Dentistry, Faculty of Dentistry, Tehran University of Medical Sciences, Tehran, Iran; ^2^Department of Dental Biomaterials, School of Dentistry/Research Center for Science and Technology in Medicine, Tehran University of Medical Sciences, Tehran, Iran

**Keywords:** Atomic force microscopy (AFM), dentin, lasers, tooth root

## Abstract

***Background:*** This study sought to assess the effect of Er,Cr:YSGG laser power on the surface roughness of lased radicular dentin using atomic force microscopy (AFM).

***Methods:*** Fifteen human radicular dentin samples were randomly divided into five groups: one control (G1, intact) and four experimental groups treated with 0.25W (G2), 0.5W (G3), 0.75W (G4) and 1W (G5) powers of Er,Cr:YSGG laser (2.78µm) at a repetition rate of 20 Hz, with a 600-µm-diameter sapphire tip. After irradiation, surface topography was analyzed by AFM using a Si probe in tapping mode. Quantitative information concerning the arithmetic average roughness (Ra) and quadratic mean roughness (Rq) was obtained from three 5×5µm areas of each sample. The data were analyzed using one-way ANOVA (P<0.05).

***Results:*** The Ra and Rq values increased in G2 and G5 and decreased in G3 and G4 groups compared to the control group. The maximum Ra and Rq values were noted in G5, which were significantly higher than the corresponding values in G3 (P<0.05).

***Conclusion:*** No direct correlation was found between Er,Cr:YSGG laser power and surface roughness of lased radicular dentin. Laser therapy with a mean power of 0.5W and 1W caused the lowest and highest surface roughness, respectively.

## Introduction

Recently implemented caries preventive measures such as the extensive marketing of fluoride-containing products and water fluoridation as well as advances in conservative restorative procedures have resulted in a reduction in the prevalence of caries among children and young adults and in increased number of natural teeth present in the oral cavity of the elderly.^1^ With tooth retention, older individuals are at a higher risk of denuded root surfaces, which might be due to periodontal diseases or mechanical traumas as a result of inappropriate plaque control measures and incorrect toothbrushing.^2^ Root surface exposure associated with factors such as frequent use of carbohydrates, poor oral hygiene, low social class and reduced salivary flow in the elderly due to systemic diseases or drug use^3-5^ often result in decreased pH and increased count of acidogenic microorganisms in the saliva and dental plaque^6,7^ and subsequently increased risk of caries in denuded root surfaces.

Recent studies have shown that over 50% of the elderly have root caries, which highlights the importance of measures and techniques to prevent caries in denuded root surfaces of the elderly.^8^

Recent studies support the efficacy of lasers for prevention of demineralization of tooth structures, particularly when sub-ablative laser parameters are applied.^9^ For this purpose, erbium lasers can be used. Erbium lasers are not suitable for removal or ablation of mineralized tissues; rather, they can be used to change the dentin structure to confer resistance to demineralization and caries.^10,11^ Changes in tooth composition occur at 100‒1200°C.^12^ On the other hand, mineral removal must be minimized from the tooth surface in order to prevent surface irregularities since surface roughness enhances plaque accumulation. Thus, knowledge about the impact of this type of laser in different powers on radicular dentin can greatly help in selection of the most ideal laser power to minimize surface roughness in lased dentin.

The effects of laser depend on the intensity of laser, distance of central beam from the object, size of the central beam and frequency and pulse of the irradiated laser.^13,14^ Er,Cr:YSGG laser is an erbium laser with a wavelengthof 2780nm. It operates with a pulsed-beam system with fiber delivery and has a sapphire tip bathed in air and water vapor mixture.^15,16^ Er,Cr:YSGG laser irradiation of enamel with 0.75-W power increases the temperature to the level that it can change the chemical composition of enamel, making it more resistant to acid dissolution. However, risk of morphological changes such as crack formation and increased surface roughness after laser irradiation has also been reported.^12,17^ Improved resistance of tooth structure to acidic challenge and increased dentinal tubule seal following irradiation of Er,Cr:YSGG laser in vitro can be translated to decreased susceptibility to caries by cariogenic bacteria in vivo and decreased tooth hypersensitivity.^18,19^

Considering all the above, this study sought to assess the impact of Er,Cr:YSGG laser power on surface roughness of lased radicular dentin using AFM. The null hypothesis was that radicular dentin surface roughness would not be significantly different after irradiation of different powers of Er,Cr:YSGG laser.

## Materials and Methods

This invitro study was conducted on 15 human third molar teeth extracted within three months. The teeth were evaluated under a stereomicroscope to ensure they did not have any cracks, defects in their structure, enamel ditching or white spot lesions. Sample size was calculated at 15 (n=3 in each of the five groups) considering alpha=0.05, beta=0.2, minimum significant difference of 50nm and standard deviation (SD) of 8nm. The teeth were immersed in 0.05% thymol solution at 37°C up to one day before the experiment when they were immersed in distilled water at 37°C.

### Sample preparation

The teeth were cut1mm below the cementoenamel junction and then 3mm apical to the previous section with a low-speed saw (Isomet, Buehler Instruments, USA) under water coolant. By doing so, a disc measuring 3mm in thickness was obtained from each root. The reason behind the selection of this particular region was that dentinal tubules in this region are oriented vertically relative to the root surface; moreover, this region is more prone to plaque accumulation and subsequent development of caries in case of gingival recession. To standardize the samples, the discs were only cut from the buccal surface of the teeth. The buccal surface of each disc was ground with 1200- and 2000-grit silicon carbide abrasive papers (Soflex, 3M ESPE, St. Paul, MN, USA) manually under water irrigation to remove the cementum and expose the dentin. Thus, we obtained a smooth surface from the buccal root surface of each tooth suitable for AFM probing. The cross-sections of dentinal tubules were visible on the surface. We tried to standardize the process of grinding of the samples in order to obtain equal thicknesses of dentinal tubules in all the samples. After polishing, the samples were immersed in distilled water for two minutes in an ultrasonic bath (Frazmehr, Isfahan, Iran) to eliminate debris. To this end, the discs were placed in test tubes containing distilled water in such a way that the tubes, mounted on a light piece of wood, floated in the ultrasonic bath. The samples were then assigned to five groups of three and stored in capped test tubes while immersed in distilled water at 37°C until laser irradiation. For easier laser irradiation, the samples were sectioned into 3-mm slices and mounted on acrylic sheets with the same thickness. Then, they were fixed at the center of a glass slide, using cyanoacrylate glue for evaluation under AFM. The five groups included a control group, which received no intervention (G1) and remained intact and four experimental groups subjected to 0.25-W(2.8J/cm^2^) (G2), 0.5-W (5.6 J/cm^2^) (G3), 0.75-W (8.4 J/cm^2^) (G4) and 1-W(11.3 J/cm^2^) (G5) laser irradiation.

### Irradiation of laser

**Figure 1:**
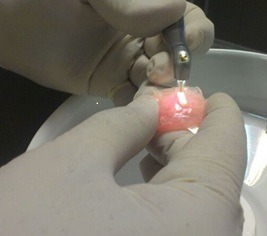
Laser irradiation on the specimen surface.

Er,Cr:YSGG laser (BIOLASE Inc.,USA) was irradiated at 2780-nm wavelength, pulse duration of 60μS and 20-Hz frequency. Laser was irradiated at 0.25-W(G2), 0.5-W(G3), (G4) and 1-W (G5) powers under water (60%) and air (80%) spray for cooling via an MZ6 glass tip (diameter of 600 μm) with swiping motion at 1-mm distance from the specimen surface in two cycles to ensure uniform irradiation of the entire surface (Figure 1). The total time of laser irradiation for each sample was approximately 30s. The samples were then stored in distilled water in capped test tubes at 37°C.

### Atomic force microscopy

**Figure 2:**
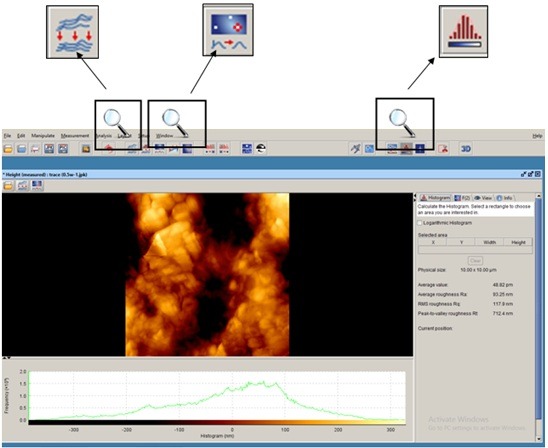
JPK software: Filters used and measurement of surface roughness.

**Figure 3:**
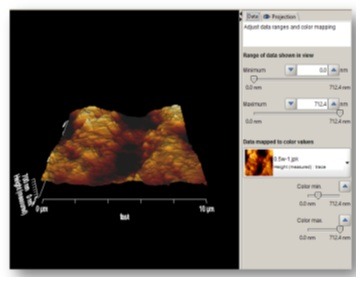
Three-dimensional view after applying the filters in JPK software.

The samples were subjected to AFM (AFM, JPK, Germany) for assessment of surface roughness and surface topography. Prior to assessment, the samples were dried with nitrogen (N_2_) gas and scanned by ACTA silicon probe. Three images measuring 5×5mm were obtained from each surface. To compensate for the possible errors during preparation of samples with abrasive papers (done to eliminate cementum and reach root dentin), all the images were corrected using two filters: subtract a polynomial fit from each scan line independency and replace value with the median of neighboring pixels (Figure 2). Next, each image was evaluated by Data Processing JPK version spm-3.4.15 for measurement of Ra and Rq surface roughness values. The Ra and Rq values were measured in three points in each area with equal distances (1μm) from each other and the mean values were reported as the mean Ra and the mean Rq for each zone. In addition, using a software feature, two-dimensional images were also evaluated three-dimensionally (Figure 3).

Statistical analysis of the data was performed using SPSS 20. One-way ANOVA was applied to compare surface roughness of the groups. P<0.05 was considered statistically significant.

**Figure 4:**
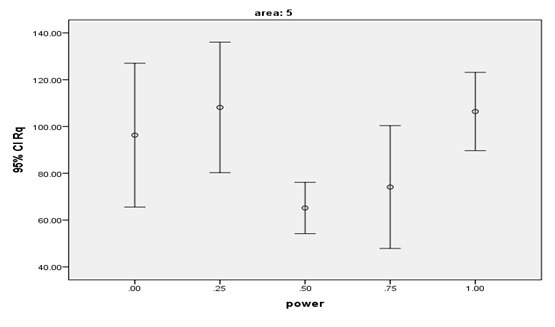
The means and 95% confidence intervals of Rq surface roughness parameter after laser irradiation.

**Figure 5:**
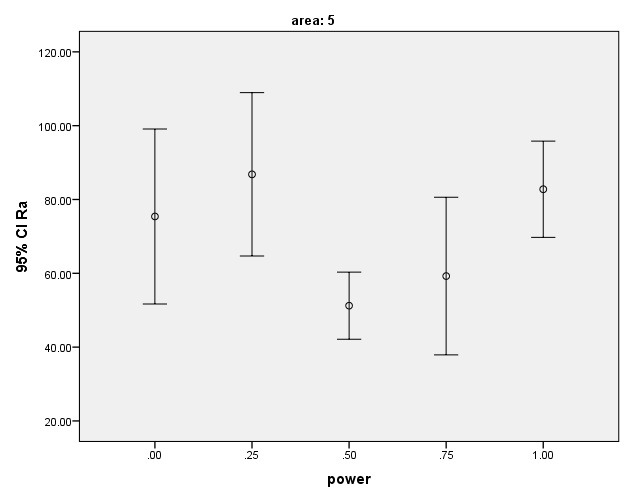
The means and 95% confidence intervals of Ra surface roughness parameter after laser irradiation.

## Results

The lowest mean Rq and Ra values were found in the laser group with 0.5-W power (G3) while the highest mean Ra and Rq values were found in the laser group with 0.25-W power(G2). The highest numerical Ra and Rq values were noted in the control group (maximum value) and the lowest numerical Rq and Ra values were noted in the laser group with 0.75-W power (G4, minimum value). Table 1 shows the mean and SD values of surface roughness in the five groups. Based on the results, surface roughness (Rq and Ra) in G3 (laser therapy with 0.5-W power) was slightly (but not significantly) lower than that in G2 (0.25-W power) and significantly lower than that in G5 (1W, P=0.003). No such significant differences were noted in other groups. The mean and 95% confidence interval of the Rq surface roughness parameter after laser irradiation are shown in Figures 4 and 5. Laser therapy with 0.5 and 0.75-W power (G3 and G4) decreased surface roughness and the lowest surface roughness value was noted after laser irradiation with 0.5-W power (G3).

**Table 1:** The means and standard deviations of surface roughness (Ra and Rq) in the five groups

**Group**	**Number of samples**	**Mean Rq (standard deviation)**	**Mean Ra (standard deviation)**
**G1**	3	96.29 (39.9)	75.4 (30.8)
**G2**	3	108.1 (2.36)	86.8 (28.7)
**G3**	3	65.1 (14.2)	51.2 (11.1)
**G4**	3	74.1 (34.1)	52.9 (27.9)
**G5**	3	106.3 (21.7)	82.7 (16.2)

G1: control group; G2: 0.25-W power; G3: 0.5-W power; G4:0.75-W power; G5: 1-W power

The mean and SD of Rq and Ra values were the lowest in G3 (0.5W, Rq=65.1±14.2nm and Ra=51.2±11.1nm) but the only significant differences were noted between these values and the corresponding values in G5 (1W, Rq=106.3±21.7nm and Ra=82.7±16.9).

The surface of samples lased with 0.25-W laser (G2) showed the highest surface roughness compared to the control group (G1). Laser therapy with 1-Wpower (G5) also increased the surface roughness but it was still lower than that in the control group.

## Discussion

This study sought to assess the relationship of the power of Er,Cr:YSGG laser with surface roughness of lased root dentin; the results showed that 0.5-Wand 0.75-W laser powers decreased surface roughness in comparison with the control group. The difference in surface roughness values was only significant between 1-Wand 0.5-Wlaser power groups.

Evidence shows that laser therapy of enamel and dentin surfaces with high power can increase their acid resistance. Improved resistance of tooth structure to acidic challenge in vitro might be translated as decreased susceptibility to caries by cariogenic bacteria in vivo.^18^ Clinical studies have reported promising results regarding the effects of laser on caries prevention in tooth structure.^20^ Increased resistance of enamel to acids was shown by Stern et al^21^ following the application of pulsed CO_2_ laser with energy densities of 13, 25 and 50 J/cm^2^. However, they also pointed to the adverse effects of laser and risk of crack formation following laser irradiation of enamel. All the three laser powers had the same efficacy for conferring resistance to enamel; however, due to less adverse effects, 13 J/cm^2^ energy density was found to be more appropriate. Later on, the efficacy of erbium lasers for conferring resistance to enamel was evaluated with promising results.^11,12^ The mechanism of action of lasers in conferring resistance to tooth structure is through decreasing the enamel solubility in acid.^22^ Freitas et al^16^ (2010) showed that Er,Cr:YSGG laser with 0.75-W power, 20-Hz frequency and 8.5 J/cm^2^ energy density was capable of conferring resistance to enamel against acidic challenges. They showed that laser increased enamel temperature to the level of melting of enamel crystals, which resulted in changes in the chemical structure and subsequently the acid solubility of enamel. However, use of optimal power and intensity of laser is extremely important since increased temperature and sudden evaporation of water in enamel minerals might lead to micro-explosions and removal of enamel minerals, which make it more susceptible to acids and enhance the occurrence of caries.^23^ Similar to its effects on the enamel, laser can confer resistance to dentin as well.

With increased life expectancy, the percentage of the elderly with oral and dental problems associated with aging such as gingival recession and crater-shaped cervical erosions, resulting in denuded root dentin, is increasing.^24^ Since dentin is more prone to acid challenges, caries in these areas occurs more frequently and progresses faster. Thus, lasers have been suggested to confer resistance to dentin. Hossain et al (2001) evaluated the effect of 5-W and 6-W Er,Cr:YSGG laser on human enamel and dentin and showed that dentin melted following laser irradiation similar to enamel and the melted dentin showed higher resistance to acid challenge than non-lased dentin.^23^ Gao et al^25^ (2006) reported changes in the crystalline structure of dentin following laser irradiation. They showed shrinkage in the a-axis dimension of hydroxyapatite, which conferred acid resistance to dent in. Aranha et al^19^ indicated that Er,Cr:YSGG laser with 0.25-W and 0.5-W powers sealed the dentinal tubules via melting and freezing, which subsequently decreases tissue permeability. In this condition, sensitivity probably decreases and the occurrence of caries reduces due to deceased penetration of acid into the underlying tissue. Another possible explanation for resistance of mineral tissues particularly dentin to acid following laser irradiation is the organic blocking phenomenon, which refers to blockage of the passage of microorganisms, toxins and nutrients due to partial denaturation of protein matrix by irradiation of laser, which delays enamel and dentin demineralization.

Apel et al^26^ (2004) indicated that Er,Cr:YSGG laser increased the microhardness of enamel surfaces and conferred resistance to acid challenge. Apel et al^11^ (2000) discussed that Er,Cr:YSGG laser was more effective than Er:YAG in decreasing enamel solubility in acid and explained that this might be attributed to greater absorption of Er,Cr:YSGG by hydroxyapatite crystals. Thus, we used Er,Cr:YSGG laser with 2780-nm wavelength, 60-μs pulse duration and 20-Hz frequency with different powers, which was irradiated on dentin with MZ6 tip. Geraldo-Martin et al^18^ (2013) showed that irradiation of Er,Cr:YSGG laser with 4.64 J/cm^2^ energy density along with water coolant further opened the dentinal tubules; they concluded that use of water coolant during laser irradiation for prevention of caries in dentin is not much effective since it results in further opening of dentinal tubules, which probably leads to faster progression of caries in dentin. Later in 2014, Geraldo-Martin et al^2^ showed that Er,Cr:YSGG laser irradiated with 8.92 J/cm^2^ energy density without water coolant created brown spots on root dentin, which were microscopically shown to be burned dentin due to overheating. They demonstrated that high power Er,Cr:YSGG laser without water coolant caused significant thermal damage in dentin and made it more susceptible to acidic challenge. Thus, laser irradiation must be necessarily accompanied by water-cooling to prevent damage to pulp and dentin; however, the amount of water must be minimal in order not to compromise the anti-caries effects of laser irradiation.^2^ Therefore, we also used a combination of water (60%) and air (80%) coolant in our study. The chosen laser powers in our study (0.25, 0.5, 0.75 and 1W) were based on a previous study by de Freitas et al,^16^ since these powers conferred optimal resistance to enamel in their study. They showed that enamel resistance caused by 0.75-W Er,Cr:YSGG laser was higher than that in groups that received fluoride therapy; other powers of laser also showed comparable effects to that of fluoride therapy. This finding is particularly important since concerns still exist with regard to fluoride therapy in young children, and an alternative method to confer resistance to the enamel would be highly appreciated. Despite the confirmed anticariogenic effects of Er,Cr:YSGG laser, they showed morphological changes following irradiation of laser, which highlights the importance of selection of an ideal power to minimize adverse morphological effects.^17^

It is noteworthy that previous studies on this topic have been mainly conducted in vitro. However, in the oral environment, bacterial plaque and biofilm have a pivotal role in the occurrence of caries. The surface roughness created by the presence of plaque causes mechanical retention and plays a key role in maintaining an acid-rich and bacteria-rich environment. In general, it can be stated that in the oral environment, it is extremely important not to increase the surface roughness while conferring resistance to enamel and dentin. According to Bollenl et al, the surface roughness threshold required for plaque retention is 0.2μm;^27^ thus, the difference in roughness of surfaces lased with different powers of laser in our study cannot significantly affect plaque retention and subsequent development of caries.

After confirming the anti-cariogenic effects of lasers, researchers attempted to find an ideal protocol of laser irradiation with maximum anti-cariogenic and minimum morphological effects. Hossain et al (2004) showed that Er,Cr:YSGG laser with 3-W power (70% air, 20% water, 33.9 J/cm^2^ energy density) increased surface roughness of dentin.^23^ Our findings revealed that Er,Cr:YSGG laser irradiation with 0.25- and 1-W powers(2.8 and 11.3J/cm^2^, respectively) increased the surface roughness compared to non-lased dentin surfaces, which was not significant; however, 0.5-W and 0.75-W powers of Er,Cr:YSGG laser decreased surface roughness compared to the control group (higher surface roughness of the control group was due to preparation of dentin with silicon carbide abrasive papers to remove cementum and denude root dentin). In our study, the only significant difference in surface roughness was noted between 0.5-W (lowest) and 1-W (highest) power groups.

Considering all the above, it seems that irradiation of Er,Cr:YSGG laser with 0.5-Wpower yields a smoother dentin surface compared to other laser powers tested in this study and can even decrease the baseline surface roughness of dentin. It appears to be due to melting of apatite crystals on the surface. Overall, it seems that change in surface roughness following the application of Er,Cr:YSGG laser with the parameters used in this study does not alter plaque retention according to the threshold set by Bollenl et al.^27^ Thus, it is advisable to select ideal laser parameters based on acid solubility. Further studies are required to confirm that laser therapy does not affect surface roughness to the level that changes plaque retention. Also, further studies are recommended to evaluatethe anticariogenic effects of a combination of fluoride therapy and laser therapy and the resultant surface roughness.

Within the limitations of this study, no direct correlation was detectedbetween Er,Cr:YSGG laser power and surface roughness of lased radicular dentin. Laser therapy with a mean power of 0.5Wand 1Wcaused the lowest and highest surface roughness, respectively.In general, it appears that change in surface roughness following the application of Er,Cr:YSGG laser with the parameters used in ourstudy does not alter plaque retention on root dentin.

## Acknowledgments

We thank our colleagues from Tehran University of Medical Sciences, Faculty of Dentistry, who provided insight and expertise that greatly assisted the research, although they may not agree with all of the interpretations/conclusions of this paper.

## Authors’ contributions

ZH, MPN and SS contributed to the concept and the design of the study. ZH prepared the samples. MPN performed the tests, and SS supervised the conduct of study. SS drafted the manuscript. All authors have contributed to the critical revision of the manuscript, and have read and approved the final paper.

## Funding

The funding for this study was provided by Tehran University of Medical Sciences.

## Competing interests

The authors declare no competing interests with regards to the authorship and/or publication of this article.

## Ethics approval

Not applicable.
